# A prospective, multicenter, randomized OCT study of early neointimal condition at first and second months after BuMA Supreme stent versus XIENCE stent implantation in high-bleeding-risk coronary artery disease patients: study protocol for a randomized controlled trial

**DOI:** 10.1186/s13063-019-3361-0

**Published:** 2019-06-07

**Authors:** Bo Li, Qin Hua Jin, Yun Dai Chen, Chang Qian Wang, Bei Shi, Xi Su, Guo Sheng Fu, Yan Qing Wu, Xu Chen Zhou, Zu Yi Yuan

**Affiliations:** 1 Department of Cardiology, Chinese Hainan hospital of PLA General Hospital, Jianglin Road, Haitangwan District Sanya, China; 2 Department of Cardiology, Chinese PLA General Hospital 28#, Fuxing Road, Haidian District Beijing, 100853 China; 3 Department of Cardiology, Shanghai 9th People’s Hospital, Shanghai, China; 4 Department of Cardiology, Affiliated Hospital of Zunyi Medical College, Zunyi, China; 5 Department of Cardiology, Wuhan Asia Heart Hospital, Wuhan, China; 6 Department of Cardiology, Sir Run Run Shaw Hospital, Zhejiang U. School of Medicine, Hangzhou, China; 7 Department of Cardiology, 2nd Affiliated Hospital of Nanchang University, Nancang, China; 8 Department of Cardiology, 1st Affiliated Hospital of Dalian Medical University, Dalian, China; 9 Department of Cardiology, 1st Affiliated Hospital of Xi’An Jiaotong University, Xi’An, China

**Keywords:** BuMA stent, Malposition, Optical coherence tomography, Stent-strut coverage, Bleeding risk, Coronary artery disease

## Abstract

**Background:**

Earlier vascular healing after drug-eluting stent (DES) implantation may reduce the incidence of late stent thrombosis and provide theoretical evidence to shorten dual antiplatelet therapy duration in patients with high bleeding risks. The BuMA supreme stent is a newly developed DES-coated with the sirolimus by using the international patent electronic grafted eG™ technology. Previous randomized trials showed that BuMA stents had better stent-strut coverage at 3-month follow-ups, which were evaluated by optical coherence tomography (OCT). However, there have been a limited number of studies that are directly evaluating the extent of neointima formation at the first and second months after stent implantation in high-bleeding-risk patients with coronary artery disease. This clinical trial is designed to demonstrate the non-inferiority of the BuMA supreme stent compared to the XIENCE stent in early neointimal formation.

**Methods/design:**

This is a prospective, multicenter, randomized trial. Forty patients will be assigned into the first-month OCT group, and another 40 patients into the second-month OCT group. The patients in each cohort will be randomized again into two groups in a 1:1 ratio, either being implanted with the BuMA Supreme stent or the Xience V/Prime/Xpedition stent. The primary endpoint is stent-strut neointimal coverage rate (%) at the first and second months, respectively. Secondary endpoints include neointimal hyperplasia area/volume, neointimal hyperplasia thickness, stent-strut malapposition rate, late lumen loss (LLL), restenosis rate, device/lesion/clinical success rate, device-oriented composite endpoints at the first and second months, stent thrombosis and other serious adverse events and bleeding events at follow-up.

**Discussion:**

The results will provide the first accurate imaging evidence on neointimal formation of the BuMA Supreme stent and the Xience stent at 1–2 months post PCI. The result should inspire further exploration and adjustment of DAPT treatments.

**Trial registration:**

ClinicalTrials.gov, ID: NCT02747329. Registered on 21 April 2016. Last updated 17 May 2018.

**Electronic supplementary material:**

The online version of this article (10.1186/s13063-019-3361-0) contains supplementary material, which is available to authorized users.

## Background

Drug-eluting stents (DES) reduce the rate of restenosis and consequent need for target lesion revascularization (TLR) compared with bare metal stents (BMS). However, impaired endothelialization increases the risk of late and very late stent thrombosis [1]. As a solution, the patient’s postoperative dual anti-platelet therapy (DAPT) treatment increases gradually from 1 month for the BMS to 6–12 months for DES post procedure, or even longer. But extension of DAPT increased the risk of bleeding, which raises another issue of how to shorten the DAPT period after the new DES implantation – which will benefit the patients with relatively high bleeding risk [2].

The BuMA stent (SINOMED, Beijing, China) is a novel, biodegradable, poly-lactic-co-glycolic acid polymer sirolimus-eluting stent (SES), with a thin-strut cobalt-chromium platform (80 μm), thin (200 nm) electro-grafting base between the polymer and stainless-steel stent strut. The BuMA Supreme SES has a pharmacokinetic polymer degradation/drug release profile where the top coat releases the drug relatively rapidly such that sirolimus release from the stents surface is nearly complete by 28 days after implantation [3]. The PIONEER trial showed that the BuMA stent did not meet the criteria for non-inferiority to zotarolimus-eluting stents (ZES) in terms of angiographic in-stent late lumen loss (LLL) at 9-month follow-up [4]. The study also showed that the BuMA SES was superior to the EXCEL SES in endothelialization at 3-month follow-up [5]. However, the earlier (≤ 2-month) endothelialization data are limited. This study can provide accurate imaging evidence on early neointimal formation of the BuMA and Xience stents, and provide evidence for further exploration and adjustment of DAPT treatment.

## Methods/design

### Study design

This is a prospective, multicenter, randomized clinical trial that should demonstrate the non-inferiority of the BuMA Supreme stent compared with the Xience stent on neointimal healing response evaluated by optical coherence tomography (OCT). In the current trial, the following devices will be used: BuMA Supreme stent – Biodegradable Polymer SES Coated Coronary Stent System manufactured by SINO Medical Sciences Technology Inc. and the Xience V/Prime/Xpedition stent – Durable Polymer everolimus-eluting Stent System manufactured by Abbott Company. A full range of commercially available guiding catheters, balloon catheters, and guidewires will be readily available.

### Patient enrollment

**Fig. 1:**
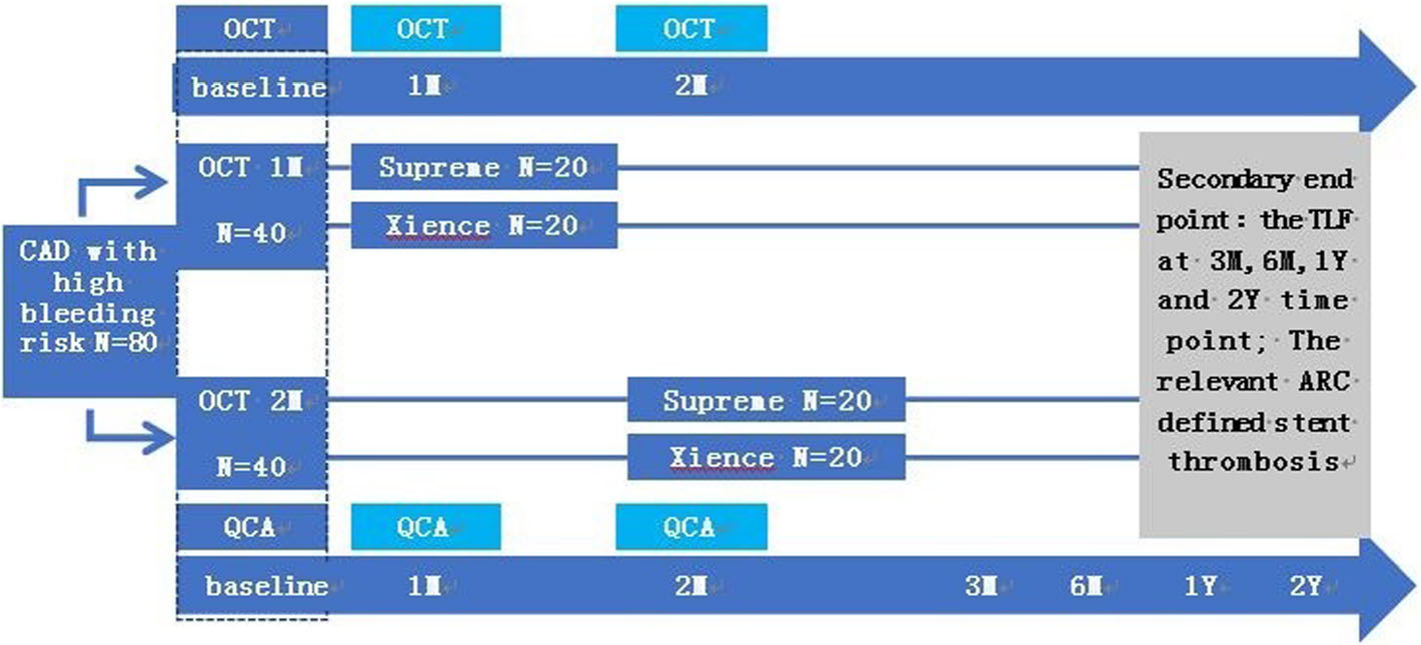
Design of study. *OCT* optical coherence tomography, *CAD* coronary artery disease

**Fig. 2:**
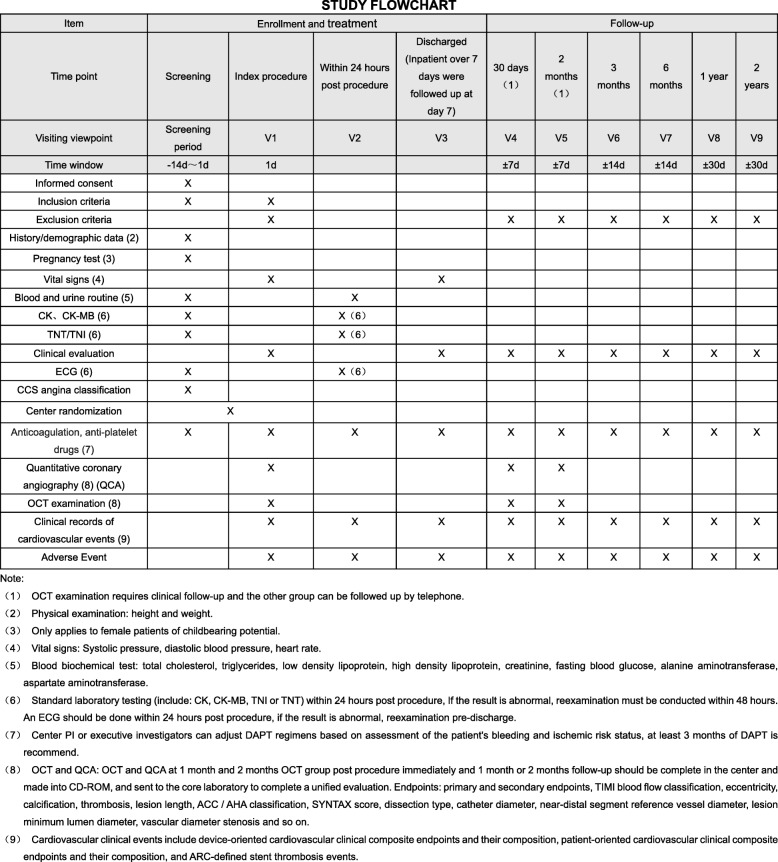
Standard Protocol Items: Recommendations for Interventional Trials (SPIRIT) Figure

Eighty coronary artery disease (CAD) patients with a high bleeding risk will be included from eight centers, the major inclusion and exclusion criteria are summarized in Table 1. Patients will be randomized into two cohorts using a 1:1 ratio, 40 patients will be assigned into a first-month OCT group. The other 40 patients will be assigned into a second-month OCT group. The patients in each cohort will be randomized again into two groups in a 1:1 ratio, either receiving the BuMA Supreme stent or the Xience stent. The process of patient recruitment, enrollment and randomization is summarized in Figs. 1 and 2 shows the Standard Protocol items: Recommendations for Interventional Trials (SPIRIT) Figure for the trial.

**Table 1:** Major inclusion and exclusion criteria

Inclusion criteria	
1. The age of patients is between18 and 85 years	
2. Symptoms and evidence of myocardial ischemia without raised troponin (e.g., stable or unstable angina, silent ischemia demonstrated by positive territorial functional study)	
3. CAD patients with a high risk of bleeding. Any one or more situation listed below can be considered the patient with high bleeding risk by the investigator [6]: a) Adjunctive oral anticoagulation treatment planned to continue after PCI b) Baseline Hb < 11 g/dl (or anemia requiring transfusion during the prior 4 weeks) c) Any prior intra-cerebral bleed at any time d) Any stroke during the past year e) Hospital admission for bleeding during the prior 12 months f) Non-skin cancer diagnosed or treated ≤ 3 years g) Planned daily NSAID or steroids for ≥ 30 days after PCI h) Planned major surgery (within 1 year) i) Renal failure (calculated creatinine clearance < 60 ml/min) j) Thrombocytopenia (< 100,000/mm^3^) k) Severe chronic liver disease (variceal hemorrhage, ascites, hepatic encephalopathy or jaundice) l) Patient with previous history of gastrointestinal bleeding m) Patient meets the following 2 or more factors: “aged female,” “diabetes,” “uncontrolled high blood pressure,” “heart failure”	
4. The patient has a planned intervention of up to two de novo lesions in different epicardial vessels	
5. Lesion(s) must have a visually estimated diameter stenosis of ≥ 70% and < 100%	
6. Reference vessel diameter (RVD) must be visually estimated ≥ 2.5 and ≤ 4.0 mm, and the lesion length must be no more than 40 mm	
7. The patient and the patient’s physician agree to the follow-up visits including angiographic follow-up and OCT controls at 1 or 2 month	
8. Written informed consent provided	
Exclusion criteria	
1. Left ventricular ejection fraction (LVEF) < 30%	
2. Patients expected not to comply with 1-month DAPT	
3. Chronic total occlusion (TIMI 0), left main lesion, intervention-required three-vessel lesions, branch vessel diameter ≥ 2.5 mm and bypass lesion	
4. Patients requiring a planned-staged PCI procedure more than 1 week after the index procedure	
5. Procedure planned to require non-study stents, or stand-alone POBA or stand-alone atherectomy	
6. Severe tortuous, calcified or angulated coronary anatomy of the study vessel that in the opinion of the investigator would result in suboptimal imaging or excessive risk of complication from placement of an OCT catheter	

*CAD* coronary artery disease, *DAPT* dual anti-platelet therapy, *OCT* optical coherence tomography *NSAID* non-steroidal anti-inflammatory drug, *PCI* percutaneous coronary intervention, *POBA* plain old balloon angioplasty

### Randomization

This is a prospective, multicenter, randomized controlled clinical trial; the central randomized method based on the computer system was used in this trial. Patients with provided written, informed consent who meet the “all inclusion and no exclusion” criteria will be enrolled into the study. After the investigator fills in the randomized application form and logs into the randomization website, the computer system will automatically assign random number and corresponding treatment groups to patients according to the patient condition. After obtaining a randomized result, the investigator should record the random number and the corresponding stent information on the randomized application form and the original case history record. Patients will be treated by the system according to the requirement of the randomized groups (the treatment group/the control group). The investigator needs to print a random result list after randomization. The clinical center should archive the randomized list and other related documents (randomized application form) together in the folder. The folder should be archived well in preparation for inspection.

### Ethical and informed consent of clinical trials

This trial will be conducted in compliance with the current Helsinki Declaration, Medical Device Clinical Trail Regulations, International Conference on Harmonization (ICH) Good Clinical Practice (GCP) guidelines and relevant national regulations. Prior to initiation of the study, the investigator will submit copies of the protocol, informed consent form and all other relevant documents to the Institutional Review Board (IRB)/Ethics Committees (EC) for its review and approval. A copy of the written IRB/EC approval must be provided to the sponsor (or designee). Any amendments to the protocol, as well as possible associated information and consent form changes, will be submitted to the IRB/EC and written approval obtained prior to implementation. Documentation of serious adverse events in the clinical trials should be submitted to the IRB/EC in time. Eligible patients (and/or the patient’s parents or the patient’s legal guardian, if applicable) should be informed about the trial and have to sign an informed consent prior to the first study procedure. The contents of the trial, purpose of the trial, expected efficacy, possible adverse events and response to adverse events of the procedures and study should be explained to the patient. Informed consents are obtained from the investigators and participants or their legally authorized representatives, and both parties save a copy.

### Medication specification

DAPT is recommended for pre-procedure and post-procedure medication in accordance with the “Chinese guideline for percutaneous coronary intervention [7]”; the proposed dose is as follows. In consideration of patients with a high-risk of bleeding, the actual loading dose may vary based on the investigator’s discretion, which will not be deemed as protocol deviation.

For patients who are already on aspirin, a maintenance dose of 100–300 mg/qds will be administered. For patients not previously exposed to aspirin, a loading dose of aspirin 300 mg will be administered as the first dose 2 h before percutaneous coronary intervention (PCI), preferably within 24 h before PCI.

For patients already on clopidogrel, a maintenance dose of 75–150 mg clopidogrel will be administered within 6 h before PCI. For patients who not previously exposed to clopidogrel, a loading dose of 600 mg of clopidogrel will be administered within 6 h before PCI. For patients already on clopidogrel for more than 72 h, no more increased load is needed.

During the procedure, patients will receive appropriate heparin according to actual situation by investigator.

Post the procedure, a subcutaneous low-molecular-weight heparin injection is given if necessary, dual anti-platelet therapy with aspirin and clopidogrel is recommended, 75 mg clopidogrel and 100 mg aspirin once a day. DAPT is recommended for at least 3 months after PCI, a maintenance of DAPT, a reduction in antiplatelet therapy or less antiplatelet treatment time using DAPT is permitted in patients by the investigator according to patient’s specific bleeding and ischemic status.

### OCT and QCA evaluation

The OCT and quantitative coronary angiography (QCA) results of this study will be evaluated by an independent core laboratory (PLA general Hospital, Beijing, China) in a blind manner. According to the standard procedure, imaging discs of the baseline procedure, baseline OCT, follow-up angiography and OCT (1 month or 2 months), and any unintended angiography will be sent to the coronary angiography core laboratory to complete the qualitative and quantitative analysis by the experienced image analyst blinded to the treatment protocol. Standard QCA methodology was used including analysis of stent and peri-stent segments defined as spanning 5 mm proximal and distal to the stent edge. Binary restenosis was defined in every segment (proximal, distal and in-stent) as > 50% diameter stenosis at follow-up. LLL was defined as the difference between follow-up and post-procedure minimal lumen diameter.

OCT parameters were calculated and defined as follows [8, 9]: neointimal thickness was measured as the perpendicular distance between the endoluminal surfaces of the neointima to the stent strut. Stent and lumen areas will be measured, and the neointimal hyperplasia area is calculated as the stent area minus the lumen area. The neointimal hyperplasia volume is calculated as the sum of the neointimal hyperplasia areas. The absence of definite neointima over the stent strut was defined as an uncovered stent strut. The distance between the inner surface of the strut reflection and the vessel wall was measured by extending the contours of the walls on the outside of the strut shadow. Stent-strut malapposition was defined as struts with detachment from the vessel wall > 138 μm for the BuMA and > 155 μm for the XIENCE (stent-strut thickness + coating thickness + OCT resolution limit of 20 μm).

### Study endpoints and clinical follow-up

The primary endpoint is the stent-strut neointimal coverage rate (%) by OCT assessment at 1 month and 2 months, respectively. Secondary OCT endpoints include neointima hyperplasia area/volume, neointimal hyperplasia thickness and the stent-strut malapposition at 1 or 2 months’ follow-up, respectively. Secondary angiographic endpoints include LLL, restenosis rate of in-stent/in-segment at 1 or 2 months’ follow-up, respectively. Secondary clinical endpoints include device/lesion/clinical success rate, device-oriented composite endpoints and its individual components, other serious adverse (MI, TLR and stent thrombosis) and bleeding events at 1 or 2, 3, 6 months, 1 year and 2 years.

Target lesion failure was defined as the composite of cardiac death, target vessel myocardial infarction (MI), and ischemia-driven TLR. MI was adjudicated according to the third universal definition of MI [10]. Stent thrombosis (definite and probable) was defined according to the Academic Research Consortium (ARC) classification. All clinical events were adjudicated by an independent Clinical Events Committee (CEC). Device success was defined as attainment of < 30% residual stenosis of the target lesion by visual assessment. Lesion success was defined as attainment of < 30% residual stenosis, TIMI 3 flow, and no residual dissection or thrombosis of the target lesion using any percutaneous method. Clinical success was defined as attainment of lesion success of the target lesion and no in-hospital major adverse cardiac event.

Angiography and OCT investigations are conducted at 1 or 2 months post PCI. Clinical follow-up visits or telephone follow-up will be conducted at the first, second, third, sixth month, or 1 to 2 years post PCI.

### Sample size of clinical trials

The study’s primary endpoint was stent-strut neointimal coverage rate (%) by OCT assessment at the first or second month. Using a two-sided α of 0.05, 20 subjects were enrolled in each group. It was assumed that 1.5 stents would be implanted in each patient with 200 stent struts (20 mm length) for each stent. Regarding a 20% drop-out rate, 4800 stent struts were estimated to be tested. Assuming a neointimal coverage rate for both the BuMA and Xience stents of 70%, and a non-inferior margin of 3.6% at the first month, stent struts would have at least 97% power to detect that the Supreme group show non-inferiority to that of the Xience group. Assuming a neointimal coverage rate for both BuMA and Xience stents of 85%, and a non-inferior margin of 2.4% at the second month, stent struts would have at least 91% power to detect that the Supreme group shows non-inferiority to that of the Xience group. Since a competition mechanism is introduced into this multicenter study, no single center will enroll more than 40% of the patients.

### Statistical analysis

Continuous variables will be described using means and standard deviations or median and range in the case of asymmetric data distribution. Categorical variables will be presented as numbers and frequencies, and comparisons will be made using the chi-square test or Fisher’s exact test as appropriate. All time-to-event outcomes will be summarized using Kaplan-Meier survival estimates and compared among treatment groups using log-rank tests. An array of prespecified and exploratory subgroup analyses of the primary and major secondary endpoints will be performed. Statistical significance will be considered at *P* < 0.05. A statistical package (SPSS 16.0) will be used for analysis. The individual will be considered as a unit of analysis.

## Discussion

PCI is widely used for patients with CAD all over the world. Compared with BMS, DES reduce the rate of restenosis from 20–35% to 10% and reduce the consequent need for TLR. However, DES increases the risk of late stent thrombosis, death and MI [11–13]. A previous study [12] reported that the incidence of definite stent thrombosis was increased by 0.53% (CI 0.44–0.64) per year, and the cumulative rate of definite stent thrombosis was 3.3%, combined incidence of definite stent thrombosis and the probable stent thrombosis was 5.7% in 4 years. At present, some studies have regarded endothelialization as one of the important factors that may reduce late thrombosis and then reduce cardiovascular events; therefore, research on DES has focused on how to promote the endothelialization. The ideal DES is to maximize the inhibition of smooth-muscle-cell proliferation and migration, minimize changes of structure and functional recovery of endothelial cell, and promote faster healing of endothelium [14, 15]. The second generation of drug-eluting stents (DES) has emerged with more biocompatible durable polymer coatings on thin-strut stents that have shown better safety than first-generation DES [16, 17]. The Biodegradable Polymer (BP)-DES, BuMA Supreme stent, was developed to prevent polymer-related adverse effects [18].

DAPT treatment increases from 1 month of the BMS to 6–12 months of DES post procedure, or even longer, which increases the risk of bleeding. Research on how to shorten the DAPT period after the new DES implantation will benefit relatively high-bleeding-risk patients. Studies showed that one out of seven to eight CAD PCI patients has a relatively high risk of bleeding, so clinicians need to evaluate bleeding and ischemic risk after DES, and adjust their DAPT treatment to obtain better clinical results [2].

In the recent 10 years, OCT has been widely used as an intravascular imaging technology to determine the morphology of the implanted stents, whereby high-resolution intravascular images can be obtained quickly – especially accurate results of stent-strut coverage in the vessel [19, 20]. For example, Ishigami K et al. observed stent-strut coverage of the first-generation sirolimus eluting stent (SES) at 9 months, 9–24 months and 25 months, respectively, and they found that only 17.6% of the SES was completely covered at 25 months. Delayed neointimal coverage was observed in patients with small blood vessels, complex lesions, high-lipid and high-calcium lesion or diabetics [21]. OCT was a reliable method for assessing the neointimal formation of stent strut post PCI, and it provided a good match to the resemblance of “optical biopsy.”

The research and development of stents is focused on the materials and coating composition of stents in China [22]. OCT is rarely used to evaluate vessel healing after new stent implantation. We have evaluated new BuMA DES strut coverage at 9 months by OCT in 2010, which showed that stent-strut coverage rate in BuMA stent was 94.3%, which was superior to that of the Endeavor stent [23]. The PANDA-III study with 2348 samples showed a statistically significant difference in the incidence of definite and probable stent thrombosis (0.7% vs. 1.4%) at 1 year, that is less stent thrombosis than occurred in the BuMA stent [24]. Moreover, in OCT follow-up at 3 months, the percentage of stent-strut coverage was significantly higher in the BuMA vs. the EXCEL group (strut level: 94.2% vs. 90.0%, *P* < 0.01) [5]. It showed that the design concept and technology of BuMA stent is good to promote early and complete healing of the neointima at a corresponding time after PCI at 3 months. The LEADERS FREE study also used large samples to compare the efficacy and safety of implantation of BMS and new DES in patients with CAD; 1-year follow-up showed that the efficacy and safety of the new DES group was superior to that of the BMS group [6, 25]. The OCT study of neointimal coverage has been reduced from 9 months to 3 months at present [5]; one purpose of this study is whether we could learn more about stent-strut coverage rate at 2 months and 1 month, which will help to conduct further study of an adjusted postoperative DAPT regimen. The evidence may provide clinicians and patients with more precise imaging evidence in DAPT regimens, especially more benefit for patients with a relatively high bleeding risk.

For BuMA stents that used international-patent, electronic-grafted (eG) technology, common problems, such as stent surface cracking and residue after drug release, are solved successfully, leading to rapid and complete endometrial healing and inhibition of late stent thrombosis. This feature is more conducive to helping patients with a relatively high risk of bleeding to adjust their DAPT treatment regimen. Based on the design of the previous generation of BuMA stents, the stent metal platform of the BuMA Supreme changed from 316 L stainless steel to L605 cobalt chromium – with thinner stent strut and better stent effects in contrast imaging and flexibility. Meanwhile, the thinner stent strut may further benefit the endothelium healing, which will bring the patients more safety benefits.

In conclusion, this is the first study to compare the strut coverage of the XIENCE stent with that of the BuMA Supreme sirolimus-eluting cobalt-chromium stent, which has a shorter drug elution on optical coherence tomography (OCT) 1 or 2 months after implantation; and it will give us accurate imaging evidence on neointimal formation of the BuMA Supreme stent and the Xience stent at 1–2 months post PCI. The result should inspire further exploration and adjustment of DAPT treatments Additional file 1.

## Additional file

Additional file 1: SPIRIT 2013 Checklist: recommended items to address in a clinical trial protocol and related documents*. (DOC 130 kb)

## Abbreviations

ARC: Academic Research Consortium
BMS: Bare metal stent
CAD: Coronary artery disease
DAPT: Dual anti-platelet therapy
DES: Drug-eluting stents
LLL: Late lumen loss
LVEF: Left ventricular ejection fraction
MLD: Minimal lumen diameter
OCT: Optical coherence tomography
PCI: Percutaneous coronary intervention
PLGA: Poly-lactic-co-glycolic acid
PTCA: Percutaneous transluminal coronary angioplasty
QCA: Quantitative coronary angiography
RVD: Reference vessel diameter
SES: Sirolimus-eluting stent
TLF: Target-lesion failure
TLR: Target-lesion revascularization
ZES: Zotarolimus-eluting stents
